# Clinical, Radiological, and Endoscopic Features of Pancreatic Pseudocyst and Walled-Off Necrosis: How to Diagnose and How to Drain Them

**DOI:** 10.3390/jcm14217818

**Published:** 2025-11-03

**Authors:** Giuseppe Dell’Anna, Salvatore Lavalle, Paolo Biamonte, Jacopo Fanizza, Edoardo Masiello, Angelo Bruni, Francesco Vito Mandarino, Paoletta Preatoni, Francesco Azzolini, Jahnvi Dhar, Jayanta Samanta, Antonio Facciorusso, Elisa Stasi, Mattia Brigida, Armando Dell’Anna, Marcello Spampinato, Marcello Maida, Sara Massironi, Vito Annese, Lorenzo Fuccio, Gianfranco Donatelli, Silvio Danese

**Affiliations:** 1 Gastroenterology and Gastrointestinal Endoscopy Division, IRCCS San Raffaele Hospital, Via Olgettina 60, 20132 Milan, Italy; fanizza.jacopo@hsr.it (J.F.); mandarino.francesco@hsr.it (F.V.M.); preatoni.paoletta@hsr.it (P.P.); azzolini.francesco@hsr.it (F.A.); massironi.sara1@hsr.it (S.M.); sdanese@hotmail.com (S.D.); 2 Gastroenterology and Gastrointestinal Endoscopy Division, IRCCS Policlinico San Donato, Piazza Edmondo Malan 2, 20097 San Donato Milanese, Italy; paolo.biamonte@grupposandonato.it (P.B.); vito.annese@grupposandonato.it (V.A.); 3 Unité d’Endoscopie Interventionnelle, Hôpital Privé des Peupliers, 75013 Paris, France; gianfranco.donatelli@unina.it; 4 Department of Medicine and Surgery, University of Enna Kore, 94100 Enna, Italy; salvatore.lavalle@unikore.it (S.L.); marcello.maida@unikore.it (M.M.); 5 Istituti Ospedalieri Bergamaschi, 24046 Bergamo, Italy; 6 Faculty of Medicine and Surgery, Vita-Salute San Raffaele University, Via Olgettina 56, 20132 Milan, Italy; masiello.edoardo@hsr.it; 7 Radiology Unit, IRCCS San Raffaele Hospital, Via Olgettina 60, 20132 Milan, Italy; 8 Gastroenterology Unit, Department of Medical and Surgical Sciences, IRCCS Azienda Ospedaliero-Universitaria di Bologna, University of Bologna, 40100 Bologna, Italy; angelo.bruni4@unibo.it (A.B.); lorenzo.fuccio3@unibo.it (L.F.); 9 Department of Gastroenterology and Hepatology, Punjab Institute of Liver and Biliary Sciences, Mohal 160062, India; jahnvi3012@gmail.com (J.D.); dj_samanta@yahoo.co.in (J.S.); 10 Department of Gastroenterology, Post Graduate Institute of Medical Education and Research, Chandigarh 160012, India; 11 Faculty of Medicine and Surgery, University of Salento, Piazza Tancredi 7, 73100 Lecce, Italy; antonio.facciorusso@unisalento.it (A.F.); elisastasi2@gmail.com (E.S.); mattiabrigida@hotmail.it (M.B.); armando.dellanna@alice.it (A.D.); 12 Endoscopy Unit, Department of Surgery, Vito Fazzi Hospital, Piazza Filippo Muratore 1, 73100 Lecce, Italy; 13 General Surgery Unit, Department of Surgery, Vito Fazzi Hospital, Piazza Filippo Muratore 1, 73100 Lecce, Italy; marcello.spampinato@gmail.com; 14 Department of Clinical Medicine and Surgery, University of Naples “Federico II”, 80138 Naples, Italy

**Keywords:** pancreatic pseudocysts (PPs), walled-off necrosis (WON), computed tomography (CT), magnetic resonance imaging (MRI), lumen-apposing metal stents (LAMS), endoscopic ultrasound (EUS)

## Abstract

Pancreatic pseudocysts (PPs) and walled-off necrosis (WON) are two distinct sequelae of acute and chronic pancreatitis, requiring accurate differentiation to guide appropriate management. Computed tomography (CT) and magnetic resonance imaging (MRI) remain essential for distinguishing PPs from WON, assessing their content, and identifying potential complications. Endoscopic ultrasound (EUS) has emerged as a key modality for both diagnosis and drainage planning, offering high-resolution imaging and the possibility of real-time aspiration. Management strategies have evolved significantly, shifting from surgical to minimally invasive approaches. Endoscopic drainage, including EUS-guided transmural drainage with double-pigtail or lumen-apposing metal stents (LAMS), has become the preferred strategy for symptomatic or infected collections. Endoscopic necrosectomy is increasingly performed for WON, providing a less invasive alternative to surgical debridement. However, patient selection and procedural techniques remain topics of ongoing debate. The aim of this review is to provide a comprehensive synthesis of current evidence regarding the diagnosis and management of pancreatic pseudocyst and walled-off necrosis. We will synthesize current evidence on diagnostic criteria, imaging modalities, and therapeutic algorithms for PPs and WON. We will discuss technical aspects, success rates, and complications associated with drainage modalities, comparing endoscopic, percutaneous, and surgical approaches. Special attention will be given to recent advancements in interventional endoscopy and their impact on patient outcomes. By integrating clinical insights with the latest literature, this review aims to provide an up-to-date reference for clinicians managing pancreatic fluid collections. A literature search was performed using PubMed, Scopus, Web of Science, and MEDLINE databases to identify relevant studies on diagnostic criteria, imaging techniques, and management strategies.

## 1. Introduction

### 1.1. Classification and Definitions of Pancreatic Fluid Collections

Pancreatic cystic lesions can be pathologically categorized into inflammatory pancreatic fluid collections (PFCs), non-neoplastic cysts, and cystic pancreatic neoplasms. Precise characterization of these lesions is essential, as it guides the appropriate management approach. In 1992, the Atlanta Symposium on Acute Pancreatitis established a classification system for PFCs, incorporating terms such as acute fluid collections, acute pseudocyst, pancreatic necrosis, and pancreatic abscess to standardize the nomenclature and improve diagnostic precision [1]. In response, the Acute Pancreatitis Classification Working Group revised the Atlanta Classification. The updated system categorizes PFCs in acute pancreatitis into four main types: acute peripancreatic fluid collection (APFC), acute necrotic collections (ANCs), pancreatic pseudocyst (PP), and walled-off necrosis (WON). APFC occurs within the first 4 weeks of interstitial edematous pancreatitis and lacks both encapsulation and necrosis [2]. The incidence among patients with acute pancreatitis (AP) has been reported to range between 40% and 50% [3]. APFCs are more frequently observed in younger patients, those with an alcoholic etiology, and individuals exhibiting elevated inflammatory markers (such as increased C-reactive protein levels at 48 h) [3]. Additional risk factors include severe disease and delayed presentation to the hospital [4]. These collections are typically homogeneous, confined to retroperitoneal fascial planes, but their composition is not well characterized. Some studies indicate they typically contain protein-rich fluid, with variable levels of pancreatic enzymes, and resemble plasma in overall composition [5]. More than half of APFCs resolve spontaneously without intervention, while the remainder may progress to PP [5,6]. ANC arises during the first 4 weeks (approximately in 5–10% of all AP cases and in over 90% of necrotizing pancreatitis) [4] and consists of a heterogeneous mixture of fluid and necrotic tissue. They lack a mature encapsulating wall and typically demonstrate heterogeneity on imaging, frequently containing loculated components and solid debris [7]. ANCs may be intrapancreatic, peripancreatic, or both and are distinguished from APFC by the presence of necrotic material rather than purely serous fluid [8]. A major complication is infection of ANCs, occurring in 20–40% of individuals with severe necrotizing pancreatitis, which is strongly associated with heightened morbidity and mortality [9]. In most cases, ANCs demonstrate temporal progression, and following approximately 4 weeks, they may develop a mature encapsulating wall, at which stage they are designated as WON. WON represents a mature, encapsulated collection of pancreatic and/or peripancreatic necrotic tissue, characterized by a well-formed inflammatory wall that typically develops more than 4 weeks after the onset of necrotizing pancreatitis [9,10]. On imaging, WON appears heterogeneous, comprising both fluid and solid components, and is distinguished from PPs by the presence of necrotic material rather than solely serous fluid [2]. Among patients with severe AP, the incidence of WON is estimated at roughly 30%. Identified risk factors for WON include elevated body mass index (BMI ≥ 25), pancreatitis secondary to endoscopic retrograde cholangiopancreatography (ERCP), and disseminated intravascular coagulation (DIC). Additional clinical predictors comprise male sex, leukocytosis at admission, and the presence of hypoenhanced pancreatic lesions on early computed tomography (CT) imaging [11]. A PP is a mature, encapsulated collection of homogeneous fluid enclosed by a well-defined wall, usually located extrapancreatically, and typically forms more than 4 weeks after the onset of AP [2]. In contrast to WON, a PP contains purely fluid without any solid necrotic material. Its wall is fibrous and non-epithelialized [12]. PPs occur in approximately 6–15% of patients following AP, with a higher incidence, up to 42%, observed in cases of acute-on-chronic pancreatitis [13,14]. The majority of PP develop within two weeks of symptom onset and are linked to more severe disease and prolonged hospitalization. Spontaneous resolution is common, particularly for small, asymptomatic cysts [15,16]. Risk factors for the development of PP following AP include a severe initial disease course, alcohol consumption, pre-existing chronic pancreatitis, delayed hospital admission, diabetes mellitus, history of biliary surgery, low hemoglobin and albumin levels, and male sex [17,18].

### 1.2. Pathophysiology of Pancreatic Pseudocyst (PP) and Walled-Off Necrosis (WON)

PPs represent a well-defined complication of inflammatory or obstructive processes involving the pancreas. These conditions result in disruption of the main pancreatic duct or its branches and subsequent extravasation of enzyme-rich pancreatic juice into the retroperitoneal space, leading to autodigestion and necrosis of the peripancreatic tissues [19,20]. In cases of parenchymal necrosis affecting the pancreatic neck or body, the distal viable remnant maintains its secretory function, continuing to release pancreatic juice. This perpetuates the inflammatory response and promotes the formation of a fluid collection that is progressively delimited by granulation tissue but lacks a true epithelial lining [21]. The organization of the fluid collection follows a temporal evolution: initially, the acute inflammatory infiltrate drives the exudative phase; subsequently, the fluid becomes separated by fibrin deposition and proliferation of granulation tissue; and ultimately, after more than four weeks, a mature and well-defined fibrous wall develops [22]. The pseudocyst, therefore, represents the “mature” stage of an acute peripancreatic fluid collection, reflecting the chronicization of the inflammatory process, with progressive replacement of necrotic tissue by fibrous connective tissue [22]. Necrosis of the peripancreatic tissues may extend to the pancreatic ducts, causing ductal disruption and direct leakage of pancreatic juice. Consequently, pseudocyst formation is closely linked to and at some point in its course requires communication with the pancreatic ductal system [23]. In this context, D’Egidio and Schein proposed a classification of PPs into three distinct types, based on the underlying etiology of pancreatitis (acute versus chronic), the anatomical configuration of the pancreatic duct, and the presence or absence of communication between the cyst and the duct [24]. Subsequently, Nealon and Walser introduced an alternative system, categorizing PP into seven types, relying entirely on pancreatic ductal anatomy and findings obtained through endoscopic retrograde cholangiopancreatography (ERCP) [25]. Specifically, type I describes a normal pancreatic duct with no communication with the cyst; in type II, the duct remains normal but a duct–cyst communication is present. Type III corresponds to an otherwise normal duct complicated by a stricture, without communication with the cyst, whereas type IV combines the stricture with the presence of a duct–cyst communication. Type V is characterized by a complete interruption (“cut-off”) of an otherwise normal duct. Finally, Types VI and VII occur in the setting of chronic pancreatitis: in Type VI, no communication with the cyst is observed, whereas in Type VII, such a communication is present [25]. In addition to necrosis and ductal rupture, obstruction due to stones, strictures, or protein plugs may also contribute by raising intraductal pressure and causing localized ductal rupture with extravasation of pancreatic juice [23,26]. WON is the mature, encapsulated sequel of acute necrotizing pancreatitis, typically arising more than four weeks after symptom onset. Severe pancreatic inflammation produces ischemic injury and necrosis of pancreatic and peripancreatic tissues; initially unencapsulated, these necrotic–fluid collections organize into a well-defined wall of granulation and fibrous tissue lacking an epithelial lining [13]. Damage-associated molecular patterns (DAMPs) released from necrotic cells drive local and systemic innate immune activation (neutrophils, macrophages) and fibroblast recruitment, segregating nonviable from viable tissue; this containment may temper systemic inflammation yet predisposes the collection to secondary infection via bacterial translocation. At the cellular level, ATP depletion, calcium overload, and reactive oxygen species disrupt membranes and perpetuate injury. The resulting demarcation and encapsulation define WON and guide management, as invasive procedures are safer and more effective once the wall has matured [10].

## 2. Materials and Methods

A comprehensive literature search was conducted using PubMed, Scopus, Web of Science, and MEDLINE databases to identify relevant studies on pancreatic pseudocyst (PP) and walled-off necrosis (WON). The search included English-language articles published up to September 2025. The following keywords and their combinations were used: “pancreatic pseudocyst,” “walled-off necrosis,” “acute pancreatitis,” “chronic pancreatitis,” “endoscopic drainage,” “lumen-apposing metal stent,” “percutaneous drainage,” and “surgical management.” Additional references were retrieved through manual screening of bibliographies from pertinent reviews and original articles. Studies focusing on diagnostic criteria, imaging modalities, endoscopic and surgical techniques, and treatment outcomes were included to ensure a comprehensive overview of current evidence.

## 3. Clinical Features and Natural History

### 3.1. Symptoms and Presentation

PP and WON may remain asymptomatic, even when large, occurring in up to 50% of WON cases. When symptoms occur, they are primarily related to the local mass effect. The most frequent manifestations include persistent abdominal pain, early satiety, nausea, vomiting, and weight loss, while obstructive jaundice due to bile duct compression and anorexia may occasionally be observed [27,28,29,30]. However, both PP and WON may progress to complications. These include rupture with ensuing acute peritonitis, fistula formation, vascular erosion with acute hemorrhage, and the development of pancreatic ascites or pleural effusion. In addition, both entities can cause hollow viscus obstruction by compression of adjacent structures such as the colon, stomach, duodenum, and common bile duct, leading to abdominal pain, vomiting, or jaundice. Infection is one of the most common complications. Most PP remain sterile, and infectious complications are uncommon; in contrast, pancreatic necrosis, particularly when it evolves into WON, carries a substantial risk of infection [8,31]. The frequency of infection in PP is generally low, estimated at approximately 5–10% in cases of acute or chronic pancreatitis [5], whereas WON is considerably more prone to infection, with reported rates reaching 30–40% among patients with pancreatic necrosis [32,33]. Infection may result from bacterial translocation from the gastrointestinal tract, secondary infection of an intracystic hematoma, or iatrogenic introduction of bacteria following procedures such as EUS-FNA or ERCP [34]. Clinically, it is important to differentiate between an infected PP, an infected ANC, and an infected WON. The detection of gas bubbles on CT imaging serves as a key indicator of infection; however, a definitive diagnosis requires aspiration of the fluid under CT or ultrasound guidance, followed by Gram staining and microbiological culture [5]. Bacteriological analysis of aspirates from infected pancreatic collections typically reveals predominantly Gram-negative intestinal bacteria, with *Escherichia coli* being the most common, followed by *Enterococcus* and *Klebsiella* [30]. In recent years, a shift toward Gram-positive organisms, including *Staphylococcus aureus* and other *Enterobacteriaceae*, has been reported. Fungal infection with *Candida* species occurs in 5–15% of cases and is associated with higher mortality and more systemic complications [35]. Evidence suggests that the routine use of prophylactic antibiotics may promote *Candida* infection and alter the bacterial profile of infected necroses, increasing the prevalence of Gram-positive pathogens [36]. Hemorrhage is an uncommon but potentially life-threatening complication, often resulting from vascular erosion by pancreatic enzymes or the formation of a pseudoaneurysm [37], with mortality rates reaching up to 40% in severe cases [23]. Hemosuccus pancreaticus is a rare, potentially fatal condition in which a pseudoaneurysm communicates with the pancreatic duct, leading to obscure gastrointestinal bleeding via the ampulla of Vater [38]. Additionally, PP can contribute to thrombosis of the portal or splenic veins. Splenic vein thrombosis may cause left-sided portal hypertension, which can manifest as isolated gastric varices [37]. The reported frequency of hemorrhage in PP is generally low, typically less than 10% [39]. In patients with WON, the incidence of hemorrhage is somewhat higher, ranging from 9% to 15% among those undergoing invasive treatment [37,40]. Spontaneous rupture occurs in less than 3% of PP, while rupture of WON represents an even rarer event [21]. In addition to perforation into the peritoneal cavity, cases have been reported of rupture extending into adjacent structures. When the rupture communicates with hollow viscera such as the stomach, duodenum, or colon, clinical manifestations may include vomiting, diarrhea, melena, hematemesis, or hematochezia [41]. Perforation into the peritoneal cavity can result in pancreatic ascites, peritonitis, or hemorrhagic shock that, clinically, is characterized by massive ascites with progressive abdominal distension, increased abdominal girth, abdominal pain, weight loss, and nausea [42,43]. Rarely, mediastinal extension produces thoracic symptoms, such as pleural effusion, dyspnea, or cough, often accompanied by high-amylase pleural effusion from a pancreaticopleural fistula [44].

### 3.2. Natural History of PP and WON

The development of PP represents the evolution of fluid collections that, following an episode of acute interstitial edematous pancreatitis, progressively organize and acquire a well-defined wall after an interval of at least four weeks from the acute event [2,45]. The reported rate of spontaneous resolution of PPs varies widely in the literature, ranging from 7% to 85%, with a median of approximately 50% [46]. This variability reflects the fact that most data are derived from retrospective surgical and autopsy studies with non-uniform diagnostic criteria. Classic prospective studies showed that spontaneous resolution is more likely for PPs present for ≤6 weeks, whereas those persisting between 7 and 12 weeks have a low likelihood of resolution and a high risk of complications, including rupture, abscess formation, and biliary obstruction, with mortality rates up to 12% [23,47]. More recent studies report higher rates of spontaneous resolution: Vitas et al. observed a 57% resolution rate in patients managed expectantly, with 38% resolving after 6 months [48], while Maringhini et al. reported 65% resolution within the first year, with smaller cysts (≤5 cm) being more likely to resolve [49]. Historically, cyst size was considered the main predictor of outcome, but recent studies indicate that large cysts (>10 cm) may have similar morbidity, mortality, and recurrence rates as smaller cysts, challenging the traditional “Rule of 6” (cysts ≥6 cm or duration ≥6 weeks warrant intervention [50]. Pancreatic duct anatomy has emerged as a more reliable predictor: patients with a normal duct (type 1) have a higher likelihood of spontaneous resolution (87%), whereas patients with obstructed or disconnected ducts (type 3) show no spontaneous resolution and respond poorly to percutaneous or endoscopic drainage [51]. These findings suggest that, in addition to size and duration, evaluation of ductal anatomy is crucial for predicting outcomes and guiding management of PPs. In contrast, the natural history of WON is less favorable compared with pseudocysts, as the presence of solid material makes complete spontaneous resolution unlikely and predisposes to late complications such as infection, sepsis, or mechanical obstruction of adjacent organs [5]. Nevertheless, it has been demonstrated that a strategy of “watchful waiting” is often safe in asymptomatic patients. In a study published by S. S. Rana et al., 70% of patients remained asymptomatic during follow-up. Among them, WON resolved spontaneously in 43%, while in 13% of patients, an increase in size from 9.3 cm to 12.6 cm was observed, though these patients continued to remain asymptomatic. Conversely, 30% of patients developed symptoms or complications, with a mean time to onset of 3.2 ± 1.3 months from the initial follow-up [52]. Similar findings were reported by Wronskí et al., who studied 16 patients with asymptomatic WON and observed that 44% remained asymptomatic during a median follow-up of 17 months (range 7–53.5 months) [33,53]. Consistently, Patra et al. followed 39 patients with WON for 6 months and found that in 77% of cases, the collection either resolved or remained asymptomatic, while only 23% required intervention [53].

## 4. Imaging and Diagnostic Approach

### 4.1. Role of Computed Tomography (CT)

**Figure 1:**
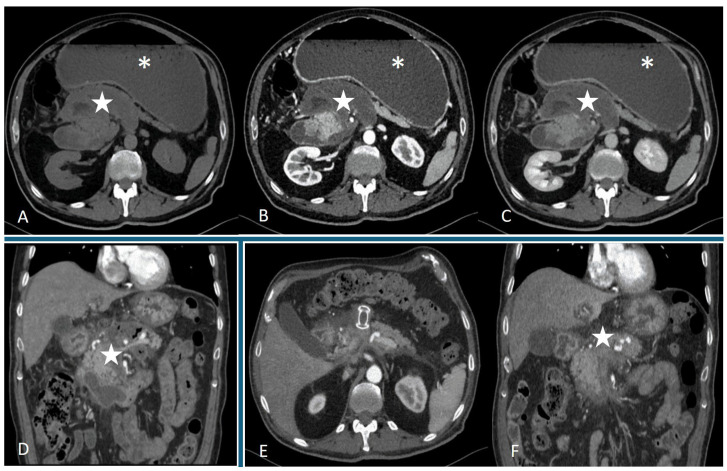
Axial unenhanced (**A**), arterial (**B**) and portal (**C**) phase CT images showing a known peripancreatic fluid collection (star) with multiloculated morphology and heterogeneous density, surrounded by thin walls, consistent with sequelae of recent necrotizing hemorrhagic pancreatitis. The collection measures approximately 12 × 8 cm in axial dimensions and 11 cm in craniocaudal extension, encasing the branches of the celiac trunk and the spleno-mesenteric-portal confluence. Associated marked gastric dilatation (asterisk) is also noted. Coronal arterial phase (**D**) showing that the collection (star) begins to exhibit imaging features consistent with the evolution toward walled-off necrosis (WON), including progressive wall maturation, internal non-liquid components such as necrotic debris, and the presence of gas bubbles. Following endoscopic drainage using a transgastric lumen-apposing metal stent (LAMS), axial (**E**) and coronal (**F**) arterial phase CT images demonstrate a marked reduction in the size of the collection (star), consistent with effective post-drainage evolution.

For pancreatitis complications, a dedicated pancreatic protocol CT is advised. A standard CT exam typically includes an unenhanced phase and contrast-enhanced phases. An initial non-contrast scan is used to detect calcifications or hemorrhage within a collection and to establish baseline attenuation. For example, an acute pseudocyst with intracystic hemorrhage might show high attenuation on non-contrast CT. Both arterial (pancreatic parenchymal) and portal venous phase imaging are usually obtained. The pancreatic phase (~35–40 s after contrast injection) optimally visualizes pancreatic perfusion and can reveal active bleeding or pseudoaneurysms, and the portal venous phase (~70 s) provides excellent delineation of fluid collections and surrounding structures [54]. Multi-phase imaging helps differentiate perfused vs. non-perfused tissue: necrotic pancreatic parenchyma will not enhance, distinguishing WON from simple fluid collections. Portal phase images also better show the extension of collections and associated venous thromboses [55]. Technical considerations include thin-section reconstructions (≤3 mm slice) for detailed analysis and multiplanar reformats. Oral contrast is generally not required; in fact, water as a negative oral contrast may be used to distend the stomach/duodenum if an endoscopic drainage route is being considered [56]. Intravenous contrast is crucial unless contraindicated (e.g., renal failure), since non-contrast CT cannot reliably distinguish solid necrotic debris from fluid due to similar attenuation [57]. CT should be performed ≥7–10 days after pancreatitis onset when evaluating for necrosis (earlier CT may underestimate necrosis extent). For follow-up of known collections, a single portal-venous phase may suffice to limit radiation, unless vascular complications are suspected [58]. On contrast-enhanced CT, pancreatic pseudocysts appear as well-circumscribed, round or oval fluid collections of homogeneous water-like attenuation, usually located in the peripancreatic region (often in the lesser sac). They are encapsulated by a thin but perceptible wall that may be enhanced due to granulation tissue. No internal soft-tissue or fat-density components are seen within a true pseudocyst [59]. In other words, the content is uniformly fluid with attenuation typically ~0–30 Hounsfield units (unless there is high-protein or hemorrhagic content, which can slightly increase attenuation). Calcification of the wall is rare; if present, alternate diagnoses like chronic abscess or cystic tumor should be considered [60]. Importantly, even minimal non-liquefied debris (e.g., strands of soft tissue or fat within the fluid) would exclude the diagnosis of pseudocyst and favor WON [61]. The surrounding pancreas in interstitial pancreatitis may be edematous but not necrotic—pseudocysts usually form after milder pancreatitis or chronic pancreatitis. CT may show signs of chronic pancreatitis (pancreatic calcifications, ductal dilation) in cases of pseudocyst arising from chronic disease. The sensitivity of CT for the diagnosis of pseudocysts is 90–100% [62]. If the pseudocyst causes mass effect, a CT scan depicts compression of adjacent structures (e.g., stomach or biliary tree) and any consequent dilation of upstream ducts. Walled-off necrosis typically appears on CT as a more complex and inhomogeneous collection compared to a pseudocyst. Since WON contains liquefied and non-liquefied necrotic material, CT often shows mixed attenuation within the encapsulated collection. Parts of the collection will be fluid-density, but there may be areas of soft-tissue density or even visible fat-density debris (from fat necrosis) floating within. The wall of a WON is usually thicker and more irregular than that of a pseudocyst [63]. WONs can be quite large, sometimes replacing a substantial portion of the pancreas or extending into remote spaces. In a landmark retrospective study, CT findings significantly associated with WON (vs. pseudocyst) included larger size, extension of the collection into the paracolic gutters or retroperitoneum, an irregular or ill-defined wall, and the presence of intralesional fat attenuation debris [64]. Additionally, the pancreas itself often shows parenchymal deformity or discontinuity on CT—for example, portions of the gland may be absent or non-enhancing, indicating prior necrosis [65]. By definition, WON forms after necrotizing pancreatitis; thus, CT may show other sequelae of necrosis, such as gas within the collection (if secondarily infected) or calcifications in chronic cases. Gas within a WON on CT is highly indicative of infected necrosis, a critical finding that usually prompts urgent intervention [66]. After IV contrast, unlike the uniform enhancement of a pseudocyst wall, a WON may have patchy wall enhancement or a multi-loculated appearance if some septa have formed. Necrotic debris does not enhance, helping to distinguish necrosis from residual viable tissue or tumor. WON collections may also cause a pronounced mass effect—e.g., compressing the stomach, colon, or inferior vena cava, as noted in some cases [67] (Figure 1).

### 4.2. Role of Magnetic Resonance Imaging

**Figure 2:**
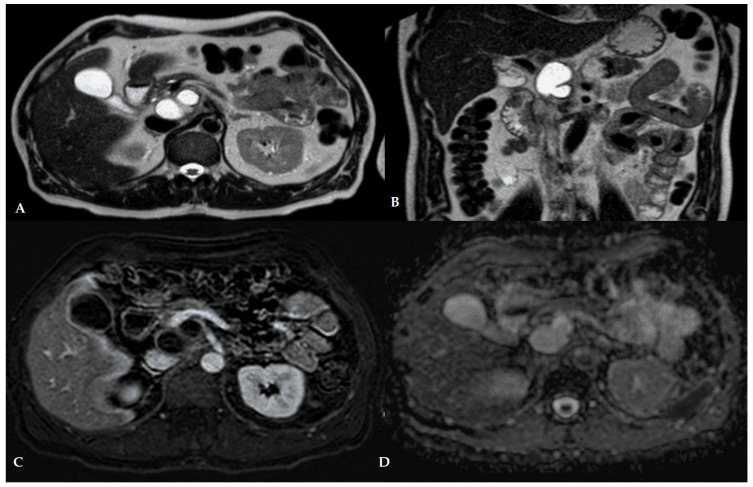
Axial (**A**) and coronal (**B**) T2-weighted MR images show a well-encapsulated, multiloculated fluid collection (arrows) with a homogeneously hyperintense T2 signal. No contrast enhancement is observed on post-contrast T1-weighted gradient-echo sequence (**C**), and no diffusion restriction is evident on the ADC map (**D**), consistent with a fluid content of serohematic/proteinaceous nature. The imaging features are compatible with a pseudocyst in a patient with a history of acute pancreatitis. The collection is located posterior to the pancreatic head and uncinate process, extending cranially into the porto-caval space.

MRI offers excellent soft tissue contrast without radiation, making it valuable for characterizing pancreatic collections. A comprehensive MRI protocol (Table 1) for pancreatic pseudocyst and WON typically includes T2-weighted sequences (with and without fat saturation), T1-weighted pre- and post-contrast images, MRCP for ductal anatomy, and often diffusion-weighted imaging (DWI) [57]. An MRI pancreatitis protocol should be performed with adequate patient fasting and hydration; administering secretin during MRCP can improve visualization of pancreatic duct leaks in select cases. Gadolinium contrast is used unless contraindicated. MRI generally requires longer acquisition time than CT; so, motion artifact control (e.g., breath-holding or respiratory gating for very ill patients) is important. Despite these challenges, MRI’s lack of radiation and superior tissue characterization make it ideal for serial follow-up imaging and detailed problem-solving in complex cases [68]. Pseudocysts on MRI mirror the CT findings in content, with additional soft-tissue detail. T2-weighted images show the cyst fluid as markedly hyperintense and homogeneous [19]. There should be no or minimal internal septations. T1-weighted images show the fluid as hypointense (dark); if the fluid is very protein-rich or hemorrhagic, it may appear slightly more intermediate on T1. The pseudocyst wall often demonstrates thin rim enhancement after gadolinium, reflecting its fibrous and vascularized inflammatory lining (Figure 2).

A smooth, thin wall (<3 mm) without nodularity is typical. No enhancing nodules should be present inside a pseudocyst—their presence would raise concern for a cystic neoplasm or intracystic tumor [69]. On MRI, MRCP sequences are particularly useful to check for communication with the pancreatic duct: up to ~50% of pseudocysts may connect to a duct branch or the main duct, especially in chronic pancreatitis or when due to ductal disruptions [70]. Identifying a duct communication (or a disconnected duct) is important for management (persistent communications may lead to recurrence if not stented or surgically addressed). MRI provides even greater clarity in depicting the components of WON. On T2-weighted MRI, WONs show a heterogeneous signal: the liquid portions are hyperintense, whereas the non-liquefied necrotic debris appears as hypointense mottled areas within the fluid [71]. This striking appearance—bright fluid with internal dark debris—is often diagnostic of WON on MR. For example, an axial T2 MR image may show a large fluid collection with dependent dark clumps or layers representing necrotic tissue. In one case series, MRI could identify necrotic debris more readily than CT, confirming that MRI is superior for differentiating WON from a simple pseudocyst [72]. On T1-weighted MRI, the necrotic material often shows intermediate to low signal (depending on composition), and hemorrhagic necrosis can cause T1 hyperintense foci. Post-contrast MRI will typically show enhancement of the WON’s fibrous wall and any internal septations, but critically, the avascular necrotic portions do not enhance. This lack of enhancement in T1 post-gadolinium helps confirm that internal pieces are dead tissue rather than, say, tumor nodules [73]. MRCP sequences might show disconnected pancreatic duct segments—a WON often forms in the setting of a ruptured duct upstream of necrosis [74]. If the main pancreatic duct is not intact across the area of necrosis, MRI will show a cutoff, indicating disconnected pancreatic duct syndrome, which has implications for recurrence risk [75]. Another advantage of MRI is visualizing complications: hemorrhage into a WON will show as T1 hyperintense fluid levels; and MRI is sensitive for venous thromboses or pseudoaneurysms that might be adjacent to the collection [68].

### 4.3. Role of Endoscopic Ultrasound (EUS)

EUS has become the diagnostic and interventional keystone in PFCs, combining a 7.5–12 MHz linear or radial transducer with direct luminal apposition. The near-field acoustic wavelength of these probes translates into sub-millimetric axial resolution, allowing for visualization of parietal layers, internal septa, and even single locules of debris that remain invisible to trans-abdominal ultrasonography or CT [5,76].

In a prospective comparison, EUS identified solid necrotic debris in 92% of collections, whereas contrast-enhanced CT detected it in only 32% (*p* < 0.001) [77,78]. When lesions were classified according to the 2012 revised Atlanta criteria, EUS differentiated pseudocyst from WON with 93–100% sensitivity and 92–98% specificity, outperforming magnetic resonance cholangiopancreatography [19,79]. This anatomical granularity guides the choice of stent calibre, number of tracts, and timing of direct endoscopic necrosectomy, thus influencing the entire step-up algorithm of severe pancreatitis. EUS depicts the thickness and vascularity of the encapsulating wall, estimates necrotic burden, and reveals ductal communication. In early retrospective cohorts, collections judged “simple” at CT were reclassified as complex in 41% after EUS inspection, prompting a change from single plastic stent drainage to Lumen Apposing Metal Stent (LAMS) or multiple gateway techniques [80]. Meta-analyses of more than 1000 patients report clinical resolution rates of 80–100% and adverse-event rates of 5–16% when EUS guidance is utilised for drainage, figures superior to blind transmural puncture and equivalent to surgery, yet with shortened hospital stay and reduced cost [77]. Haemorrhagic complications stem chiefly from arterial pseudo-aneurysms formed by enzymatic digestion of the splenic, gastroduodenal, or short gastric arteries. Although the overall prevalence of vascular events after acute pancreatitis is approximately 5%, pseudo-aneurysms complicate 4–10% of mature pseudocysts and WON and carry a mortality of up to 90% if rupture occurs undetected [81,82]. Colour and power Doppler integrated in EUS delineate turbulent flow within the aneurysmal sac and, crucially, its relation to the intended puncture route; lesions as small as 2 mm that escaped cross-sectional imaging have been diagnosed exclusively by this modality [83]. Real-time vascular mapping before drainage has therefore become mandatory, especially when deploying LAMS, which have been associated with a three-fold higher rate of delayed arterial bleeding compared with double-pigtail stents. Beyond diagnosis, EUS offers a minimally invasive alternative to treat pseudo-aneurysms. Series combining platinum coils with cyanoacrylate or thrombin report technical success exceeding 90% and durable haemostasis without surgical conversion. The largest dedicated cohort analysed eight patients in whom visceral pseudo-aneurysms (splenic in 62.5%, gastroduodenal in 25%, short gastric in 12.5%) were first excluded from the circulation by radiological angio-embolisation and then followed, within 72 h, by EUS-guided transmural drainage of the associated PFC [82,84]. Median collection diameter was 6.5 cm; clinical and radiological resolution occurred after 3.9 ± 2.5 weeks, and no re-bleeding or collection recurrence was recorded during 24 months of follow-up. These data validate a combined sequential strategy—angio-embolisation to secure the artery, EUS to drain the cavity—that is now recommended by expert consensus when active bleeding coexists with mass effect [85,86]. Moreover, infection develops in roughly one-third of WON and is the principal determinant of mortality. Radiological gas is neither sensitive nor specific, and empirical broad-spectrum antibiotics favour the emergence of multidrug-resistant flora. Under EUS guidance, a 19- or 22-gauge needle can sample ≥1 mL of turbid fluid through the gastric wall without traversing skin or peritoneum. In a multicentre observational study, microbial colonisation was demonstrated in 59% of aspirates; culture results mandated modification in antimicrobial therapy in 78% of cases, a change that would have been missed had empiric protocols been maintained [19]. Earlier work by Negm et al., in 2013, confirmed that peri-pancreatic aspirates obtained at EUS altered antibiotic choice in 65% of patients and shortened fever duration compared with CT-guided aspiration [87]. Complication rates of EUS-Fine Needle Aspiration (FNA) in this setting remain below 1%, limited to transient intracavitary bleeding that resolves spontaneously, and no cases of needle-track seeding have been reported. In this context, European evidence-based guidelines recommend mandatory EUS evaluation before any transluminal intervention and prescribe repeat Doppler assessment whenever LAMS are in situ for longer than four weeks to anticipate delayed arterial erosions [79,86,88,89]. Furthermore, when infection is suspected but not radiologically proven, aspiration rather than empirical escalation is advised to curb antimicrobial resistance.

## 5. Management Strategies

### 5.1. Conservative Management

Contemporary guidelines converge on a watch-and-wait strategy for uncomplicated PP and WON, yet the precise thresholds that justify observation differ subtly across societies. The 2024 American Society for Gastrointestinal Endoscopy (ASGE) guideline, updating earlier AGA statements, defines conservative care as appropriate when the cavity is ≤6 cm, has existed for <4 weeks, contains <10% solid debris, and is unaccompanied by pain, obstruction, infection or rapid growth; in their pooled analysis only 18% of such collections subsequently required intervention, and mortality was nil [90]. The 2019 ESGE guideline adopts a nearly identical 6 cm diameter cut-off but extends the “benign neglect” window to 8 weeks provided the wall is maturing and the main pancreatic duct (MPD) is intact; in prospective European cohorts this strategy achieved spontaneous involution in 64% of AP-related PP and 22% of chronic pancreatitis (CP)-related PP, with a conversion-to-drainage rate of 28% driven chiefly by late pain or biliary compression [91]. In contrast, the 2024 Chinese multicentre consensus—drawing on a registry that included a higher proportion of alcoholic and hypertriglyceridaemic pancreatitis—permits surveillance of cavities up to 8 cm if serial magnetic resonance cholangiopancreatography (MRCP) at 2-week intervals documents a shrinking trend and C-reactive protein remains <30 mg/L; they reported radiological disappearance in 45% of sterile WON and 71% of pseudocysts at a median 5.6 months [92]. The novel Endoscopy and Ultrasound (i-EUS) Italian consensus, synthesising data from 2839 endoscopically managed cases, harmonises these positions by recommending observation for any sterile collection <7 cm, regardless of type, during the first 4 weeks of disease, provided there is no MPD disconnection, gastric outlet obstruction, biliary stenosis, visceral pseudoaneurysm or systemic inflammatory response; failure of diameter reduction by >20% at week 4 triggers interdisciplinary review [19]. Across all guidelines, clinical trajectory rather than size alone dictates escalation. The pooled risk of secondary infection during surveillance is 0.7% per patient-month (ASGE), rising to 1.9% when the cavity exceeds 10 cm (ESGE). Predictors of delayed intervention include paracolic extension (odds ratio [OR] 4.04), ≥30% solid necrosis (OR 3.8) and disconnected duct syndrome (OR 5.7), records derived from the Dutch PANTER, German GEPARD and Italian i-EUS registries [79,93,94]. Conversely, spontaneous resolution is favoured by strict oral feeding resumption within 48 h, absence of multiorgan failure, and persistent serum albumin >35 g/L—variables that, in multivariate models, halve the probability of drainage at 6 months. When intervention becomes unavoidable, all societies advocate endoscopic ultrasound-guided drainage as the first choice, yet they underscore that premature drainage (<4 weeks) outside life-threatening infection or compartment syndrome confers no mortality benefit and doubles stent-related adverse events. Thus, conservative management remains the cornerstone, provided rigorous imaging (contrast CT or secretin-MRCP) and biochemical monitoring are maintained every 2–4 weeks until definitive regression or clear-cut indications for therapy emerge.

### 5.2. Endoscopic Drainage

#### 5.2.1. EUS-Guided Transmural Drainage

**Figure 3:**
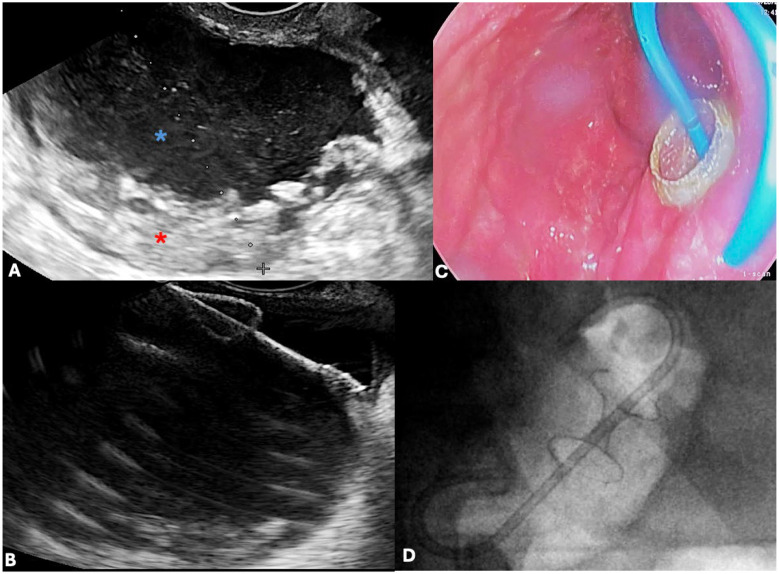
Endoscopic Ultrasound-guided Fluid Collection Drainage (EUS-FCD) of Walled Off Necrosis (WON). (**A**) WON with necrotic hypercoic part (red asterisk) and hypoechocic liquid part (blue asterisk). (**B**) EUS view of the distal flange of electrocautery-enhanced Lumen Apposing Metal Stent (ec-LAMS) into the WON. (**C**) Endoscopic and radiologic (**D**) view of ec-LAMS with coaxial double pigtail plastic stent.

The first description of endoscopic drainage of PFCs dates back to 1989, when conventional endoscopic transmural drainage (EGD-TD) was introduced. This approach relied on the endoscopic identification of a gastric or duodenal bulge caused by the underlying collection, which was then punctured to create a cystogastrostomy tract. Following dilation, plastic stents were inserted to maintain patency [95]. The advent of endoscopic ultrasound-guided transmural drainage (EUS-TD) significantly expanded the therapeutic landscape by enabling real-time visualization of the collection, identification of adjacent vasculature or necrotic components, and fluid aspiration to confirm correct positioning [96]. As a result, technical and clinical success rates were substantially improved; in fact, a randomized prospective study reported a 100% success rate with EUS-TD compared with 33% for conventional drainage (*p* < 0.001) [97]. Consequently, current international guidelines now recommend EUS-TD as the preferred endoscopic approach for managing PFCs [7,98]. For many years, double-pigtail plastic stents (DPPSs) were the standard device used in EUS-TD. These stents are inexpensive, widely available, easy to place and remove, and associated with a favorable safety profile [96]. However, their narrow caliber necessitates the placement of multiple stents and repeated dilations to achieve an adequate cystoenterostomy, which is often required for direct endoscopic necrosectomy (DEN). While DPPSs are highly effective for pseudocyst drainage (85.1–90.8%), their performance in WON is suboptimal, with clinical success rates ranging only between 30.8% and 52.1% [99]. To overcome these shortcomings, fully covered self-expandable metal stents (FC-SEMSs) were introduced. Their larger lumen permits improved drainage of solid material and lowers the risk of occlusion, and a prospective case series by Talreja et al. [100] reported a 95% clinical success rate in PFC drainage. Nonetheless, the tubular design and lack of anchoring mechanisms resulted in high rates of migration, occasionally leading to serious adverse events such as perforation or bleeding, which limited their widespread adoption. LAMSs were developed to address these issues. These devices, made of braided nitinol and fully silicone-covered, are characterized by a short, “dumbbell-shaped” structure with bilateral flanges at a 90° angle, designed to anchor the stent and prevent migration [101,102]. This design not only stabilizes the device but also provides a large-diameter conduit that facilitates drainage of solid necrotic debris and easy endoscopic access to the cavity for DEN [103]. The subsequent introduction of electrocautery-enhanced LAMSs (EC-LAMSs, HOT-AXIOS, Boston Scientific, Marlborough, MA, USA) further simplified the procedure by allowing for single-step access to the PFC, eliminating the need for tract dilation, guidewire exchange, or fluoroscopic guidance (Figure 3). A study by Bekkali et al. [104] demonstrated shorter procedure times with EC-LAMS compared to standard LAMS, supporting their adoption as the preferred option in clinical practice.

#### 5.2.2. Double-Pigtail Plastic Stents (DPPSs) vs. Lumen-Apposing Metal Stents (LAMS)

International guidelines diverge in their recommendations regarding the preferred stent for endoscopic drainage of PFCs. The ESGE states that both DPPSs and LAMSs are acceptable options [98] whereas the AGA guidelines favor LAMSs as the preferred device [7]. This divergence largely stems from the heterogeneity of available studies, which provide conflicting results regarding efficacy, safety, and cost-effectiveness. As a result, no strong universal recommendation can yet be made (Table 2).

##### Efficacy Outcomes Across Studies

Bang et al. conducted a randomized controlled trial including 60 patients with WON, who were assigned to EUS-TD using either LAMS (n = 31) or DPPSs (n = 29) [105]. The primary endpoint was the total number of procedures required to achieve treatment success, defined as both symptom resolution and complete WON clearance on CT scan at 6 months. The results showed no significant difference between groups in the number of procedures required (median 2 for LAMS vs. 3 for plastic, *p* = 0.192), treatment success, adverse clinical events, readmissions, LOS, or overall costs. LAMS placement was associated with a significantly shorter procedure time (15 vs. 40 min, *p* < 0.001), but also with higher procedural costs (USD 12,155 vs. USD 6609, *p* < 0.001) [105].

In a multicenter prospective cohort study by Boxhoorn et al., patients with infected necrotizing pancreatitis who underwent an endoscopic step-up approach with LAMS (n = 53, across 16 hospitals over 27 months) were compared with a cohort of 51 patients previously enrolled in the randomized TENSION trial, in which the same step-up approach was performed using DPPSs [106]. The study protocols were otherwise identical, allowing for a direct comparison of outcomes. The primary endpoint was the requirement for endoscopic transluminal necrosectomy (ETN), while secondary outcomes included mortality, major complications, length of hospital stay, and healthcare costs. The need for ETN was observed in 64% of patients treated with LAMS and 53% of those treated with DPPSs, with no statistically significant difference even after adjustment for baseline characteristics (OR 1.21; 95% CI, 0.45–3.23). Secondary outcomes likewise showed no significant differences between groups. Mortality rates, length of hospitalization, and total healthcare costs were comparable (mean cost difference –€6348; 95% CI –€26,386 to €10,121) [106]. Karstensen et al. performed a single-center randomized controlled trial in patients with very large WON (>15 cm), randomized to DPPSs (n = 22) or LAMS (n = 20) [107]. The primary endpoint was the number of necrosectomies required to achieve clinical success, defined as both clinical and CT resolution. Secondary endpoints included technical success, adverse events (AEs), length of hospital stay, and mortality. Technical success rates were high and comparable (100% DPPS vs. 95% LAMS, *p* = 0.48), as were clinical success rates (95.5% vs. 94.7%, *p* = 1.0). The mean number of necrosectomies (2.2 vs. 3.2, *p* = 0.42), hospital stay (43 vs. 58 days, *p* = 0.71), and overall mortality (4.8%) did not differ significantly between groups [107]. Conversely, a large multicenter retrospective study by Chen et al., involving 189 patients (LAMS n = 102, DPPS n = 87), found significantly higher clinical success with LAMS (80.4% vs. 57.5%, *p* = 0.001) [108]. This outcome was defined as a reduction in the WON size to ≤3 cm within 6 months without the need for PD or surgery. Technical success was similar (100% vs. 98.9%, *p* = 0.28). The need for PD was comparable (13.7% vs. 16.3%, *p* = 0.62), but surgical intervention was required more often in the DPPS group (16.1% vs. 5.6%, *p* = 0.02). Furthermore, recurrence rates were lower with LAMS (5.6% vs. 22.9%, *p* = 0.04 after ≥6 months follow-up). The authors concluded that, compared with DPPS, LAMS are associated with higher clinical success, shorter procedure time, a reduced need for surgery, and a lower recurrence rate [108]. A cost-effectiveness model further supported LAMSs, suggesting that their higher upfront cost may be offset by improved clinical efficacy [109]. In summary, although LAMSs offer technical advantages and may be more effective in complex cases of large WON requiring DEN, plastic stents remain a valid and cost-effective option, especially for less complicated PFCs. Ultimately, the choice between DPPSs and LAMSs depends largely on the clinical scenario, the presence of necrotic debris, device availability, and the expertise of the endoscopist [110].

##### Safety Outcomes and Adverse Events

AE profiles varied across studies. In the RCT by Bang et al., LAMS were associated with significantly higher stent-related AEs compared with DPPSs (32.3% vs. 6.9%, *p* = 0.01) [105]. Events in the LAMS cohort included buried stents (n = 2, one with massive bleeding), severe gastrointestinal bleeding from pseudoaneurysms (n = 3), and LAMS-induced biliary strictures from duodenal deployment (n = 3). Conversely, only two stent-related AEs occurred with DPPSs (stent migrations), while clinical AEs (bleeding from a pseudoaneurysm, aspiration pneumonias, pulmonary embolism) were rare and manageable. Notably, most stent-related complications in the LAMS group occurred ≥3 weeks post-intervention. Following an interim audit, the study protocol was modified to include CT imaging at 3 weeks with LAMS removal in cases of resolved WON, which reduced the difference in AEs between the two groups [105]. In the study by Boxhoorn et al., safety outcomes were comparable. Bleeding requiring intervention occurred in 9% of patients treated with LAMS versus 22% in the DPPS group, although the difference did not reach statistical significance (RR 0.44; 95% CI 0.16–1.17) [106]. In Karstensen et al., procedure-related AEs occurred in 5 patients (12%; 4 DPPS and 1 LAMS, *p* = 0.35) [107]. In the DPPS group, there were two cases of retroperitoneal perforation during stoma dilatation and one case of periprocedural sepsis, all successfully managed conservatively or with antibiotics. In the LAMS group, one patient developed sepsis after necrosectomy, which resolved with antibiotics. Finally, in the large multicenter retrospective study by Chen et al., overall AE rates were similar (9.8% LAMS vs. 10.3% DPPS, *p* = 0.90) [108]. Reported AEs included bleeding (n = 10), peritonitis (n = 4), perforation (n = 2), stent misdeployment (n = 1), and others (n = 2). Severe AEs were observed in 2.0% of the LAMS group and 6.9% of the DPPS group (*p* = 0.93). Stent dysfunction included migration (2.9% LAMS vs. 6.9% DPPS, *p* = 0.20) and occlusion (20.6% LAMS vs. 12.6% DPPS, *p* = 0.15) [108].

##### Indications and Technical Considerations

The ESGE guidelines identify confirmed or presumed infected pancreatic necrosis (IPN) as the main indication for performing invasive procedures, including radiological, endoscopic, or surgical technique [98]. Additional indications include symptomatic collections, particularly when large WON cause abdominal pain, gastric compression with nausea, vomiting, early satiety, or obstruction of the gastrointestinal or biliary tract [111]. The optimal timing for endoscopic drainage of pancreatic fluid collections remains a matter of ongoing debate. Current guidelines advocate postponing intervention for approximately four weeks, allowing for encapsulation and maturation of WON to facilitate safer access and reduce the risk of complication [98]. However, this strategy has been increasingly challenged. Several studies have explored the outcomes of earlier drainage. Trikudanathan et al. retrospectively evaluated 193 patients with necrotizing pancreatitis who underwent drainage—primarily for infection—using LAMS or other stent types [87]. Among the 76 patients treated early (<4 weeks) and the 117 treated later (≥4 weeks), the overall risk of complications was not significantly different. Reported rates of stent occlusion or infection, bleeding, perforation, and fistula formation were 40% vs. 33% (*p* = 0.36), 10.5% vs. 10.3% (*p* = 0.95), 0% vs. 6% (*p* = 0.044), and 32.9% vs. 20.5% (*p* = 0.054), respectively [87]. However, early drainage was associated with higher mortality (13% vs. 4%) and a greater need for rescue open surgery (7% vs. 1%), findings most likely attributable to the increased severity of disease in the early intervention cohort [87]. Conversely, a meta-analysis suggested higher mortality associated with early interventions, without a reduction in complication rates or improvement in clinical success [77]. More recent evidence, however, has tempered these concerns. A systematic review and meta-analysis by Ramai et al. reported no significant differences in technical or clinical success, AEs, or mortality between early (182 patients, 28.9%) and delayed (448 patients, 71.1%) EUS-TD, although hospital stay was prolonged in the early group [112]. Similar findings have been echoed by the i-EUS group. Their consensus does not discourage early EUS-TD of infected necrosis in critically ill patients unresponsive to antibiotics [113]. These evidences suggest that strict adherence to the “4-week rule” may not always be necessary [113]. Another technical aspect under discussion is the coaxial placement of a DPPS through a LAMS. This adjunctive strategy has been hypothesized to reduce LAMS-related AEs such as bleeding, occlusion, and migration by preventing flange friction and food impaction.

Several studies have reported conflicting findings. Puga et al. observed a reduction in overall AEs, particularly bleeding, with adjunctive DPPS placement, whereas AbiMansour et al. found no significant differences in clinical success (75.9% LAMS vs. 69.6% LAMS + DPPS, *p* = 0.34) or overall AEs (15.7% vs. 15.7%, *p* = 0.825) [114,115]. The first RCT addressing this issue was conducted by Vanek et al., including 67 patients who underwent LAMS placement with (*n* = 34) or without (*n* = 33) coaxial DPPS at two tertiary centers [116]. Technical success was 100% in both arms. However, the global AEs rate was significantly lower in the LAMS + DPPS group compared with LAMS alone (20.7% vs. 51.5%, *p* = 0.008). Stent occlusion was the most frequent AE and occurred significantly less often with coaxial DPPSs (14.7% vs. 36.3%, *p* = 0.042). Although the failure rate of the index method (29.4% vs. 48.5%, *p* = 0.109) and mortality with LAMSs in place (2.9% vs. 12.1%, *p* = 0.197) were numerically lower in the DPPS group, these differences did not reach statistical significance. Thus, while the Vanek RCT suggests a potential protective role, larger randomized trials are needed before coaxial DPPS placement can be universally recommended in clinical practice. The multiple transluminal gateway technique (MTGT) represents another strategy for managing complex or multiloculated WON. By creating multiple transmural fistulas with either DPPSs or LAMSs, this technique enhances the drainage of extensive necrotic cavities [116]. Initial reports showed superior treatment success compared with single-gateway drainage [117,118]. Varadarajulu et al. compared MTGT with the single gateway technique (SGT) [118]. In their series, treatment success was achieved in 11 of 12 patients (91.7%) treated with MTGT versus 25 of 48 patients (52.1%) undergoing SGT. Importantly, the need for endoscopic necrosectomy and surgery was significantly reduced in the MTGT group, highlighting its potential role in improving clinical outcomes while reducing the burden of more invasive interventions [118]. Subsequent reports have supported the use of MTGT in specific scenarios, particularly when the necrotic cavity is large (>12 cm), multiloculated, or anatomically complex [116]. Similarly, the Orlando Protocol proposed by the Varadarajulu group suggested a modified MTGT strategy for WON >10 cm associated with disconnected pancreatic duct syndrome (DPDS): this included LAMS placement to facilitate direct endoscopic necrosectomy (DEN) access, combined with two additional DPPSs proximally, which were left indwelling indefinitely to prevent recurrence [118]. ESGE guidelines recommend considering MTGT in cases of extensive or multiloculated WPN, or when single-gateway drainage is insufficient [97]. Finally, the choice of LAMS diameter is an important determinant of procedural and clinical outcomes. Both 15 and 20 mm LAMS are widely used, with larger diameters being associated with higher rates of clinical success and fewer necrosectomy sessions [102]. Importantly, multivariable analysis demonstrated that clinical success was six times more likely with 15 mm stents compared with 10 mm stents [102]. Evidence also supports the use of 20 mm LAMS in selected patients with large collections and extensive necrosis. In fact, the 20 mm stent caliber was associated with a reduced number of necrosectomy sessions compared with 15 mm stents [119].

#### 5.2.3. Endoscopic Necrosectomy for WON

##### Direct Endoscopic Necrosectomy (DEN) Techniques

**Figure 4:**
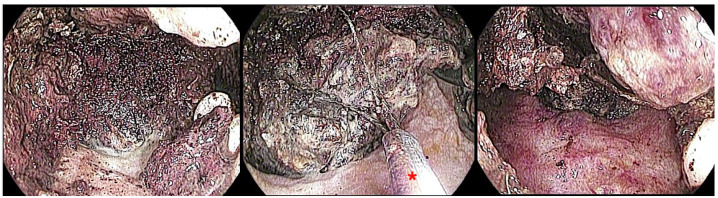
Endoscopic figures sequence of Direct Endoscopic Necrosectomy by Dormia Basket (red asterisk).

Following EUS-TD, a conventional forward-viewing endoscope can be advanced through the cystogastric or cystoenteric tract to access the WON cavity directly and perform mechanical debridement of necrotic material. DEN typically involves a combination of suctioning debris through the working channel, removal with snares, baskets, or forceps, and repeated irrigation of the cavity (Figure 4).

Predictors of the need for DEN include the presence of ≥30% solid necrosis, large collections (>10 cm), or paracolic extension [120]. The need for multiple DEN sessions is well documented. In a meta-analysis including 8 studies with 233 patients, the mean time from onset of acute pancreatitis to endoscopic necrosectomy was 7 weeks, and the weighted mean diameter of WON was 12.9 cm [121]. The pooled analysis showed that a mean of 4.09 procedures (95% CI 2.31–5.87%) were required for complete resolution, with an overall clinical success rate of 81.8% (95% CI 76.7–86.4%). Recurrence of necrotic cavities or pseudocysts after DEN occurred in about 10.9% of cases (95% CI 7.3–15.1%). These data highlight that DEN is often a multistep process, particularly in patients with large or complex collections [121]. A novel tool for predicting DEN complexity is the QNI classification system proposed by Baroud et al., which stratifies WON based on the number of abdominal quadrants involved (“Q”), the percentage of necrosis (“N”), and the presence of infection (“I”) [122]. Patients with low QNI scores (≤2 quadrants, ≤30% necrosis, no infection) required significantly fewer DEN sessions, had shorter hospitalization, and lower mortality, while those with high QNI scores (≥3 quadrants, >30–60% necrosis, or superinfection) needed more DEN interventions (2 vs. 1, *p* = 0.001), had longer times to resolution (≈80 vs. 48 days, *p* = 0.02), and showed higher mortality [122]. This classification may therefore be useful to identify patients who will likely benefit from an early and more aggressive endoscopic approach, including upfront necrosectomy. Importantly, the caliber of the LAMS plays a role: collections drained with 20 mm LAMS were associated with a reduced number of DEN sessions compared to 15 mm LAMS, supporting the concept that larger fistulas promote more effective passive drainage and facilitate debridement [122].

Regarding devices, DEN is still limited by the lack of dedicated instruments, with operators often using tools designed for other indications such as snares, baskets, nets, or forceps. Recently, innovative devices have been developed to simplify and accelerate debridement:

- The EndoRotor^®^ Powered Endoscopic Debridement System, capable of simultaneously cutting and aspirating necrotic debris, avoiding repeated scope passages [123];
- The OTSG Xcavator™, a large grasper mounted at the endoscope tip, can remove up to 1 cm^3^ of necrosis per pass while preserving the working channel for irrigation [124];
- The Necrolit^®^ system, combining a stiff monofilament snare with a Dormia basket in a single catheter, is designed to reduce procedure time.

These devices have shown encouraging preliminary results in small case series, although robust prospective trials are needed to validate their safety and efficacy.

Finally, the timely removal of LAMS is a crucial step in the DEN strategy: due to the increased risk of stent-related bleeding after 3–4 weeks, current guidelines recommend LAMS retrieval within 4 weeks of placement, with replacement by double-pigtail plastic stents when prolonged drainage is required [97]. This scheduled reassessment also provides an opportunity to evaluate WON resolution and to decide on the necessity of additional DEN sessions.

##### Step-Up Approach vs. Primary Necrosectomy

Both the ESGE and AGA guidelines recommend performing DEN only in patients with persistent clinical symptoms, organ failure, or elevated inflammatory markers after adequate drainage, explicitly discouraging its execution during the index drainage procedure [7,97]. While DEN is the preferred modality for debridement of necrotic collections, especially for its high clinical success rates and potential to avoid surgery, it is generally reserved as a step-up intervention when drainage alone proves insufficient [102]. Indeed, up to 50% of patients achieve resolution with drainage alone, particularly when treated with large-caliber LAMS, which often facilitate spontaneous extrusion of necrotic material [95]. A multicenter expert survey confirmed this strategy, showing that 85% of endoscopists discouraged DEN during the index procedure, favoring an “on-demand” rather than a scheduled approach for subsequent necrosectomies [112]. The timing of DEN remains a subject of ongoing debate. A pivotal multicenter study by Yan et al. evaluated 271 patients with WON who underwent EUS-TD with LAMS, divided into two groups: immediate DEN at the time of stent placement versus delayed DEN (≥1 week after stenting) [125]. The technical success of LAMS placement was 100% in both groups. Clinical success did not significantly differ (91.3% vs. 86.1%; *p* = 0.3), but importantly, the mean number of necrosectomy sessions required for resolution was significantly lower with immediate DEN (3.1 vs. 3.9; *p* < 0.001). On multivariate analysis, performing upfront DEN was an independent predictor of WON resolution with fewer sessions (OR 2.3; *p* = 0.004) [125]. These findings were corroborated in the DESTIN randomized trial by Bang et al., a multicenter RCT conducted across six tertiary centers, which directly compared upfront necrosectomy with a step-up approach in patients with infected necrotizing pancreatitis [126]. Seventy patients were randomized, and all underwent LAMS placement (20 mm diameter). The primary endpoint—number of reinterventions required to achieve treatment success—was significantly lower in the upfront necrosectomy group (median 1 [IQR 0–1]) than in the step-up group (2 [IQR1–4]; difference –1, 95% CI −2 to 0; *p* = 0.0027). Importantly, mortality (0% vs. 6%) and disease-related adverse events (32% vs. 48%; *p* = 0.17) did not differ significantly between the two groups, indicating that upfront necrosectomy was not associated with a higher risk, but did allow for a reduction in the overall procedural burden [126]. Most recently, a single-center RCT from Denmark specifically addressed patients with very large WON (>15 cm), comparing an accelerated approach (necrosectomy during the index procedure and repeated as needed) with a step-up protocol [127]. The trial was prematurely terminated for ethical concerns after randomizing 25 patients due to a clear benefit in the accelerated group. The composite primary outcome (death, major complications, or LOS > 58 days) occurred in only 8.3% of patients undergoing accelerated treatment versus 61.5% in the step-up group (ARR 53.5%, RR 0.14; *p* = 0.011). Notably, major complications were absent in the accelerated arm (0.0% vs. 46.2%; *p* = 0.015), and median LOS was nearly halved (32.5 vs. 68.5 days; *p* = 0.039). These results strongly suggest that upfront necrosectomy may not only reduce the procedural burden but also significantly decrease morbidity and hospital stay in patients with extensive necrosis. Taken together, current evidence suggests that while the guideline-endorsed step-up strategy remains the standard, upfront necrosectomy at the time of drainage may safely reduce the number of reinterventions needed, particularly in selected patients with infected, well-encapsulated WON. This approach is gaining traction, though careful patient selection and procedural expertise remain essential to minimize risks [127].

##### Safety Outcomes and Complications

DEN, while being less invasive than surgery, is not devoid of risks. It remains an invasive procedure in which serious AEs, including death, may occur. Reported AEs rates vary widely across studies, ranging from 7.2% to 36%, and encompass both mild and life-threatening complications [125,128]. Notably, the timing of necrosectomy—whether immediate or delayed—does not appear to significantly affect the incidence of AEs, as demonstrated in comparative studies (7.2% vs. 9.4%, *p* = 0.81) [125].

Bleeding represents the most common complication, with a pooled incidence of approximately 18% in a systematic review of 455 patients [128]. It may occur either during the necrosectomy procedure itself or after LAMS placement, as the friction between the stent and the collapsing cavity wall can erode adjacent vessels. The splenic and gastroduodenal arteries are most frequently involved, with pseudoaneurysm formation reported in 4.3–6.4% of patients with necrotizing pancreatitis [129]. Preventive measures include performing a CT scan two to three weeks after drainage and removing the LAMS if the collection has resolved, as well as carefully evaluating the cavity for the presence of visible vessels, with pneumatic stent dilation proposed as a strategy to assess the WON content. Management depends on severity: mild bleeding can be controlled endoscopically with epinephrine injection, clips, coagulation graspers, or tissue adhesives, whereas severe hemorrhage requires interventional radiology embolization [112].

Stent dislodgement is another potential complication, typically occurring during necrosectomy maneuvers when the LAMS is inadvertently captured and displaced. Although rare, successful endoscopic replacement techniques have been described [130]. Perforation, reported in up to 4% of cases, usually arises from the separation of the gastrointestinal wall from the collection during LAMS dilation [128]. Stable patients without peritonitis may be managed conservatively, whereas perforation with leakage of necrotic material into the peritoneal cavity requires surgical intervention. A rare but potentially fatal complication is air embolism, resulting from inadvertent communication between gas and the bloodstream during debridement [131]. The use of CO_2_ insufflation, which is absorbed more rapidly than air, is strongly recommended to reduce this risk; however, fatal gas embolism has nonetheless been reported even with CO_2_. Prompt recognition is essential if sudden cardiovascular or respiratory instability occurs during DEN. Overall, meticulous patient selection, timely LAMS removal, preferential use of CO_2_ insufflation, thorough vascular evaluation of the WON cavity, and performance of DEN by experienced endoscopists are key strategies to minimize the risk of severe complications.

### 5.3. Percutaneous Drainage

#### 5.3.1. Indications and Technical Approach

Percutaneous drainage (PD) is a core component of modern management of pancreatic fluid collections and infected necrosis. It is indicated when infected necrotizing pancreatitis is proven or strongly suspected—e.g., gas within a collection on CT/MRI, or persistent sepsis despite antibiotics; ongoing or impending organ failure in the setting of necrosis; symptomatic mass effect/complications, such as gastric outlet or biliary obstruction, abdominal compartment physiology, or pain; and when endoscopic access is not feasible (e.g., collections with dominant extension into the paracolic gutters, pelvis, or deep retroperitoneum). Contemporary guidelines endorse cross-sectional imaging (contrast-enhanced CT or MRI/MRCP) to confirm maturity (>4 weeks) and define anatomy before intervention, with delayed drainage favored unless clinical deterioration mandates earlier action [132]. In randomized data (POINTER Trial), immediate drainage offered no advantage over postponed drainage and led to more interventions, underscoring the “delay-if-you-can, drain-if-you-must” principle [132]. Procedural planning uses contrast CT (and often MRI/MRCP) to map the collection, detect vascular hazards/pseudoaneurysms, and choose a retroperitoneal route whenever possible to minimize peritoneal contamination; a transperitoneal path is reserved for selected scenarios [133]. Ultrasound or CT guidance is standard; image-fusion or combined endoscopic/US/CT “triple guidance” techniques can improve targeting in complex WON [134]. After antibiotic coverage and correction of coagulopathy, access is typically gained with a Seldinger technique and placement of a pigtail catheter. Many centers start with 8–12 Fr and upsize (e.g., to 14–20+ Fr) over 48–72 h if output is thick/particulate or clinical response is inadequate; multiple catheters are common for multiloculated cavities. Continuous or intermittent saline lavage can aid clearance; several groups use double-catheter irrigation systems and staged tract dilation when planning percutaneous endoscopic debridement or Video-Assisted Retroperitoneal Debridement (VARD) as the next step [135]. Drains are managed to low continuous suction or gravity with scheduled flushes; serial clinical and laboratory reassessment and interval imaging (usually CT) guide upsizing, repositioning, or placement of additional drains. Failure criteria prompting escalation typically include persistent sepsis/organ failure and inadequate catheter output despite optimization [133].

#### 5.3.2. Limitations and Role in Step-Up Strategies

PD is less effective for solid necrotic burden, which often requires debridement (endoscopic or surgical). Important complications include pancreaticocutaneous fistula, hemorrhage, visceral injury, and catheter malfunction; reported external fistula rates vary widely (≈15–25% in series/meta-analyses) and are higher than with primary endoscopic internal drainage. These drawbacks are central to the preference for endoscopic transmural routes when anatomically feasible [7,91]. PD is the first rung of the minimally invasive step-up approach for infected necrotizing pancreatitis: drain and debride (if needed). In the landmark PANTER RCT, step-up (initial PD followed by minimally invasive retroperitoneal necrosectomy when necessary) reduced major complications or death compared with primary open necrosectomy [136]. The TENSION RCT later showed that an endoscopic step-up (EUS-guided drainage ± necrosectomy) and a surgical step-up had similar rates of death/major complications, with fewer pancreatic fistulas and shorter hospital stay after the endoscopic route; long-term follow-up confirmed these advantages in fistula formation and reinterventions [137]. POINTER demonstrated that postponed drainage (after wall maturation) is as safe as immediate drainage and requires fewer invasive procedures. Collectively, high-quality evidence supports PD as an initial decompressive/sepsis-control maneuver and a bridge to definitive debridement—either endoscopic necrosectomy when a safe luminal route exists or VARD when a retroperitoneal tract is established. Summarizing, favor PD first when collections are remote from the stomach/duodenum (paracolic gutters, pelvis), when airway/anesthesia risks argue against prolonged endoscopy, or when urgent sepsis control is needed and imaging shows no safe endoscopic window. Conversely, favor an endoscopic-first step-up when a large WON abuts the gastric/duodenal wall and internal drainage can minimize fistula risk. Dual-modality drainage (DMD)—PD plus endoscopic transmural drainage—can shorten hospitalization and external drainage duration in extensive, compartmentalized WON [7,138].

## 6. Comparison of Different Treatment Approaches

The management of WON has experienced a significant transformation over the past twenty years. Traditionally, the standard treatment for infected necrosis involved immediate open necrosectomy, a procedure with high morbidity and complication rates exceeding 50% and mortality rates nearing 40% in some studies [139,140]. To reduce these risks, minimally invasive surgical necrosectomy techniques were developed, aiming to lower physiological stress and reduce procedure-related complications [139]. The key PANTER trial later validated the “surgical step-up” (SSU) approach, where patients were initially treated with percutaneous catheter drainage, with minimally invasive necrosectomy reserved for those who did not respond [136]. Concurrently, endoscopic methods surfaced as alternatives, starting with transluminal drainage and later evolving into DEN. Comparative trials showed that endoscopic treatments caused less post-procedure inflammation, resulted in fewer complications such as pancreatic fistulas, and offered additional benefits like shorter hospital stays, better quality of life, and lower indirect costs compared to minimally invasive surgical necrosectomy. These findings gradually shifted clinical practice toward an “endoscopic step-up” (ESU) strategy [137] (Table 3).

### 6.1. Endoscopic Drainage vs. Percutaneous Drainage

#### 6.1.1. Efficacy Outcome

Across prospective, retrospective, and pooled evidence, endoscopic drainage consistently outperforms PD on measures of clinical success, efficiency, and long-term durability. In the 8-year prospective cohort, Wan et al. enrolled 147 patients with PFCs, ultimately evaluating 62 who underwent EUS-TD and 67 who underwent PD [141]. When stratified by collection type, outcomes diverged. Among pancreatic pseudocysts (n = 59), EUS-TD achieved a higher rate of initial clinical success than PD (94.9% vs. 65.0%, *p* = 0.003), while overall clinical success rates were 97.4% vs. 85%, respectively (*p* = 0.072). Reintervention was required in only 2.6% of EUS-TD patients compared with 35% of PD patients (*p* = 0.004). In patients with WON (n = 70), technical success was 100% in both groups. Clinical success was similar between ED and PD (65.2% vs. 72.3%, *p* = 0.54), yet important secondary outcomes highlighted the superiority of the endoscopic route. Initial success—defined as resolution without the need for secondary interventions—was much higher in EUS-TD (65.2% vs. 17%, *p* < 0.001). PD was associated with a significantly higher incidence of reintervention (59.6% vs. 21.7%, *p* = 0.003) and adverse events (85.1% vs. 26.1%, *p* < 0.001). Moreover, residual necrosis occurred only in the PD group (two cases), while no residual lesions remained after EUS-TD. Mortality was lower in EUS-TD (3 deaths) than PD (6 deaths, *p* = 0.011), despite similar recurrence rates (two cases each) [141].

These findings are mirrored in the 14-year cohort by Keane et al. where 164 patients were analyzed: 109 underwent EUS-TD and 55 underwent PD. Among the EUS-TD group, 35% were treated for WON, with the remainder having pancreatic pseudocysts [142]. EUS-TD demonstrated robust outcomes. Treatment success rates were high and comparable between pseudocysts and WON (72% vs. 67%, *p* = 0.77), with a median post-procedural hospital stay of only 4 days. Reintervention was required in 31% of pseudocyst patients and 21% of WON patients, with no significant difference [142]. By contrast, patients undergoing PD alone experienced substantially inferior outcomes. Overall treatment success was 31% in PD vs. 70% in EUS-TD (*p* < 0.001). Although PD was associated with slightly lower rates of procedural failure (1% vs. 7%) and adverse events (1% vs. 10%), the long-term clinical trajectory strongly favored EUS-TD. Patients treated with PD required significantly more interventions (median 3.3 vs. 1.8), had a much higher prevalence of residual collections (67% vs. 21%), and more frequently required surgical rescue (11% vs. 4%) [142].

In a larger real-world Indian cohort, Samanta et al. (n = 218 WON) again demonstrated higher clinical success with EUS-TD (92.1% vs. 64.6%, *p* < 0.0001) [143]. Importantly, the solid-component burden influenced outcomes: PD performed worst when >40% solid debris was present, whereas EUS-TD achieved the best results when collections contained <40% solid material, highlighting a mechanistic advantage of endoscopic necrosectomy in cases with liquefied or semi-solid debris [143].

These single-center and multicenter cohorts have been complemented by high-level evidence from the systematic review and meta-analysis by Khizar et al., which included 17 studies and 1170 patients (543 EUS-TD and 627 PD) [144]. Technical success was high and comparable between EUS-TD and PD (OR 0.81, 95% CI 0.31–2.1, I^2^ = 0%), reflecting the feasibility of both procedures. However, clinical success—the primary outcome defined as sepsis resolution and avoidance of surgical rescue—strongly favored EUS-TD (OR 2.23, 95% CI 1.45–3.41, I^2^ = 28%, *p* = 0.0002). Subgroup analysis revealed that this advantage was particularly pronounced in WON, where EUS-TD nearly tripled the odds of clinical success compared with PD (OR 2.74, 95% CI 1.56–4.81, I^2^ = 50%, *p* = 0.0005). In contrast, in postoperative PFCs (POPFC), no significant difference emerged (OR 1.38, 95% CI 0.72–2.63). EUS-TD was also associated with a markedly shorter hospital stay, with a pooled mean difference of –15.02 days (95% CI −20.18 to −9.86), a finding of clear clinical and economic significance. Reintervention rates were substantially lower with EUS-TD (OR 0.25, 95% CI 0.16–0.40), underscoring its efficiency as a one-stop therapeutic modality compared with PD, which often requires multiple catheter exchanges or adjunctive surgical procedures. Most strikingly, mortality favored EUS-TD, with a pooled OR of 0.24 (95% CI 0.09–0.67), suggesting a nearly four-fold survival benefit over PD [144].

#### 6.1.2. Safety and Adverse Events

Safety outcomes across cohorts demonstrate nuanced differences between modalities. In Keane et al., procedural AEs were more frequent with ED than PD (10% vs. 1%), but the overall clinical trajectory still favored ED due to fewer residual collections, fewer interventions, and fewer conversions to surgery [142]. Reported complications included stent migration, pneumoperitoneum, gastrointestinal bleeding, esophageal perforation, pneumothorax, and aspiration pneumonia. Importantly, no patients died within 30 days of ED. In contrast, Wan et al. observed that the adverse events were more common with PD (65% vs. 33.3%, *p* = 0.02), including drainage tube repositioning and one in-hospital death [141]. Length of hospital stay was significantly shorter in EUS-TD (11.9 ± 4.8 days) compared with PD (34.6 ± 14.7 days, *p* < 0.001). Similarly, Samanta et al. reported higher mortality and more adverse outcomes overall with PD, while ED was associated with more rapid and frequent resolution of organ failure [143]. Certain complications are more characteristic of PD. Rana et al. documented pancreatic fistula formation in 22% of patients undergoing PD, compared with none in those treated with EUS-TD (*p* = 0.02) [148]. This complication reflects the risk of external pancreaticocutaneous fistulas inherent to percutaneous routes, which can prolong morbidity and necessitate additional interventions. The Khizar meta-analysis provides a broader context: while pooled adverse event rates were numerically lower with EUS-TD (OR 0.62, 95% CI 0.27–1.39), the difference did not reach statistical significance overall, and heterogeneity across studies was moderate (I^2^ = 72%) [144]. However, subgroup analysis clarified this pattern: in WON, EUS-TD was associated with significantly fewer adverse events (OR 0.37, 95% CI 0.17–0.81, I^2^ = 64%, *p* = 0.01), while in POPFC, no significant difference was observed (OR 2.47, 95% CI 0.92–6.60). Stent migration and occlusion were infrequent and comparable between groups (OR 0.61, 95% CI 0.10–3.88) [144]. Taken together, the totality of evidence strongly supports endoscopic drainage as the preferred first-line modality for WON, providing higher rates of clinical success, fewer reinterventions, shorter hospital stay, and lower mortality compared with percutaneous approaches, while maintaining a comparable safety profile. The superiority of EUS-TD is most evident in collections with necrotic debris (WON), where endoscopic transluminal access allows for direct necrosectomy, a therapeutic capability unavailable to PD.

### 6.2. Endoscopic Drainage vs. Surgical Approaches

#### 6.2.1. Efficacy Outcome

Endoscopic strategies generally match or surpass surgical approaches in achieving resolution of WON while reducing procedural burden and healthcare utilization. In the landmark randomized trial by Bakker et al. (n = 22 analyzed), endoscopic transgastric necrosectomy not only attenuated the systemic inflammatory response—as reflected by significantly lower post-procedural IL-6 levels—but also translated into superior clinical outcomes, with only 20% of patients in the endoscopic group reaching the composite endpoint of major complications or death, compared with 80% in the surgical arm [145]. These findings were confirmed in a larger single-center RCT by Bang et al. (n = 66), in which an ESU approach demonstrated clear superiority over minimally invasive surgical step-up [146]. The composite endpoint of major complications or death occurred in only 11.8% of patients managed endoscopically versus 40.6% in the surgical cohort (*p* = 0.007). Endoscopy also led to improved quality-of-life scores at 3 months and a significant reduction in overall treatment costs, underscoring the patient-centered and economic benefits of endoscopic therapy [146].

The multicenter TENSION trial, conducted by the Dutch Pancreatitis Study Group, further refined this comparison [147]. This was a randomized, parallel-group superiority trial across 24 hospitals, including all Dutch university centers, enrolling 98 patients with infected necrotizing pancreatitis. Participants were randomized to an endoscopic step-up or surgical step-up approach. The primary endpoint was a composite of death or major complications within 6 months, defined as new-onset organ failure (cardiovascular, pulmonary, or renal), bleeding requiring intervention, perforation of a visceral organ, enterocutaneous fistula, or incisional hernia. At 6 months, the composite outcome occurred in 43% of the endoscopic group versus 45% of the surgical group (*p* = 0.88), showing no difference in the primary endpoint. However, endoscopy still conferred several secondary benefits: shorter hospital stays, reduced need for intensive care, and markedly fewer pancreatic fistulas—efficacy signals that matter for patient recovery and resource utilization [147]. Long-term data were provided by the ExTENSION study, which reevaluated 83 survivors from the original TENSION cohort after a mean follow-up of 7 years [137]. The primary composite outcome of death or major complications occurred at similar rates in both groups (53% endoscopic vs. 57% surgical, RR 0.93, 95% CI 0.65–1.32, *p* = 0.688). Nevertheless, meaningful differences emerged in secondary endpoints: endoscopy was associated with fewer pancreaticocutaneous fistulas (8% vs. 34%, RR 0.23, 95% CI 0.08–0.83) and fewer late reinterventions (7% vs. 24%, RR 0.29, 95% CI 0.09–0.99). Pancreatic insufficiency (both endocrine and exocrine) and long-term quality of life did not differ significantly, but endoscopy was associated with lower rates of recurrent acute pancreatitis and fewer complications in patients with disconnected pancreatic duct syndrome. Taken together, the ExTENSION trial highlighted that while long-term survival and global complication rates were comparable, endoscopy conferred lasting benefits in terms of fistula prevention, reintervention rates, and management of ductal disruption [137].

Finally, the recent network meta-analysis by Tan et al., including 21 studies and 1850 patients, synthesized available evidence and ranked upfront endoscopic necrosectomy as the most effective strategy across 11 outcomes, including lowest mortality, complication rates, and new-onset organ failure [149]. The EUS approach also performed favorably, showing lower risks of fistula formation and visceral perforation compared with surgical strategies. In contrast, upfront open necrosectomy consistently ranked lowest across all outcomes. Cost-effectiveness analyses reinforced the clinical data, demonstrating that endoscopic approaches yielded the highest net monetary benefit and enabled earlier recovery by an average of 34.1 days [149].

#### 6.2.2. Safety and Adverse Events

Safety findings across trials consistently favor endoscopy over surgery. In the trial by Bakker et al., endoscopic necrosectomy virtually eliminated new-onset multiple organ failure (0% vs. 50%) and substantially reduced the incidence of pancreatic fistulas (10% vs. 70%) [145]. Similarly, Bang et al. reported significantly fewer major complications in the endoscopic arm, with strikingly no enteral or pancreatic-cutaneous fistulas compared with 28.1% in the surgical cohort (*p* = 0.001) [146]. While mortality did not differ significantly (8.8% vs. 6.3%), the mean number of major complications per patient was substantially lower with endoscopy (0.15 ± 0.44 vs. 0.69 ± 1.03, *p* = 0.007) [146]. In the TENSION trial, although the overall composite endpoint of death or major complications did not differ, the safety profile again favored endoscopy [147]. Patients in the endoscopic arm had shorter hospital stays and fewer pancreatic fistulas—outcomes with immediate impact on postoperative recovery and long-term morbidity [147].

The ExTENSION long-term follow-up provided further granularity on late adverse events [137]. Over 7 years, endoscopy was associated with a significantly lower incidence of pancreaticocutaneous fistulas, both early and persistent, compared with surgery (8% vs. 34%). Persistent fistulas, when they occurred, were often linked to disconnected pancreatic duct syndrome, and most resolved with additional endoscopic or transpapillary drainage rather than surgical reintervention. Importantly, patients in the surgical arm required substantially more secondary interventions to address late complications, many of which ultimately involved endoscopic drainage. Regarding pancreatic function, endocrine and exocrine insufficiency developed at similar rates in both groups over the long term (≈40–60%), reflecting the underlying disease process rather than the intervention itself [137]. However, recurrent acute pancreatitis occurred less frequently in the endoscopic arm (19% vs. 37%), particularly among patients with ductal disruption, suggesting a potential protective effect of maintaining ductal access and drainage via endoscopy [137]. Overall, the cumulative evidence underscores that endoscopic strategies not only achieve equivalent or superior short-term efficacy compared with surgery, but also provide enduring safety advantages—especially in reducing fistula formation, minimizing reinterventions, and preventing recurrent disease in patients with ductal disruption.

#### 6.2.3. Impact on Patient Morbidity and Length of Hospital Stay

One of the most consistent findings across the comparative studies is the clear impact of the drainage modality on overall morbidity and hospitalization. In the cohort reported by Wan et al., patients treated with EUS-TD experienced a significantly shorter hospital stay (11.9 ± 4.8 days) compared with those managed by PD (34.6 ± 14.7 days, *p* < 0.001) [141]. A similar pattern emerged in the large series by Keane et al., where hospitalization was dramatically prolonged in the PD group, with a median stay of 42 days versus just 4 days for EUS-TD patients [142]. Notably, individuals treated with PD also required an external drain for a median of 56 days (range 3–651), a factor that not only prolonged recovery but also likely impaired quality of life, underscoring the long-term morbidity associated with this approach. Rana et al. further confirmed this advantage in the early phase of AP, showing that endoscopic transluminal drainage achieved resolution in nearly half the time compared with percutaneous catheter drainage (30.9 vs. 61.9 days), while also reducing the need for surgical rescue and preventing the formation of external pancreatic fistulae [148]. Samanta et al. corroborated these findings, demonstrating not only faster resolution of organ failure (3 days vs. 10 days; *p* < 0.0001) but also a reduction in mortality in patients undergoing EUS-TD compared with PD, particularly in the subgroup with infected WON [143]. When compared with surgical approaches, the benefits of endoscopic drainage on morbidity are even more pronounced. Bakker et al. showed that endoscopic necrosectomy markedly reduced the systemic inflammatory response, prevented new-onset organ failure, and lowered the risk of pancreatic fistula formation compared with surgical necrosectomy [145]. Bang et al. quantified this morbidity benefit, reporting fewer major complications per patient, significantly lower rates of pancreatic or enteral fistula, and improved quality of life in the endoscopic step-up group, alongside a substantial reduction in healthcare costs [146]. Although the TENSION trial did not demonstrate a mortality advantage of endoscopy over surgery, the reduction in pancreatic fistula formation and shorter hospitalization still favored the endoscopic approach [147].

These consistent findings were further reinforced by the recent network meta-analysis by Tan et al., which highlighted that both upfront endoscopic necrosectomy and the endoscopic step-up approach were associated with the shortest hospital length of stay and the most favorable morbidity profiles [149]. Importantly, the meta-analysis also demonstrated that endoscopic strategies were the most cost-effective, offering not only superior clinical outcomes but also earlier recovery—on average 34 days sooner—when compared with surgical or percutaneous modalities. Collectively, these results emphasize that endoscopic interventions, when technically feasible, represent the approach most likely to minimize patient morbidity, shorten hospitalization, and optimize resource utilization in the management of infected necrotizing pancreatitis.

## 7. Future Perspectives and Conclusions

Over the past decade, the landscape of endoscopic management of PFCs has been transformed by the introduction of dedicated devices. LAMS have significantly improved technical feasibility and efficacy by providing a wide-caliber conduit for drainage and endoscopic necrosectomy [150,151]. Continuous refinements in stent design—such as anti-migration features, electrocautery-enhanced delivery systems, and the possibility of multiple tract creation—are expanding the therapeutic potential of endoscopic drainage (ED). Furthermore, innovations in intraprocedural imaging, including the integration of contrast-enhanced EUS and novel endoscopic platforms for necrosectomy, are expected to enhance safety and reduce the need for repeat procedures. While EUS-TD remains the preferred first-line modality, hybrid approaches are increasingly recognized as valuable in selected scenarios. Dual-modality drainage (DMD), combining PD with EUS-TD, has emerged as a promising option for large, complex, or anatomically challenging WON. Ross et al. first reported on 117 patients with symptomatic and infected WON managed with DMD, demonstrating 0% pancreaticocutaneous fistula, no need for surgical necrosectomy, and no procedure-related deaths [152]. This technique allows for redirection of pancreatic juice into the gastrointestinal tract, reducing the morbidity associated with external fistulas, while PD provides access to undrainable retroperitoneal or pelvic extensions and facilitates bedside lavage of necrotic debris. Although EUS-TD with LAMS and endoscopic necrosectomy is now the standard of care, DMD remains an essential adjunct in patients with disconnected pancreatic duct syndrome (DPDS) or with inaccessible necrotic cavities. Current ESGE guideline recommend considering the combined approach in WON extending to the paracolic gutters or pelvis, where exclusive endoscopic access is limited [97].

## 8. Future Directions

**Figure 5:**
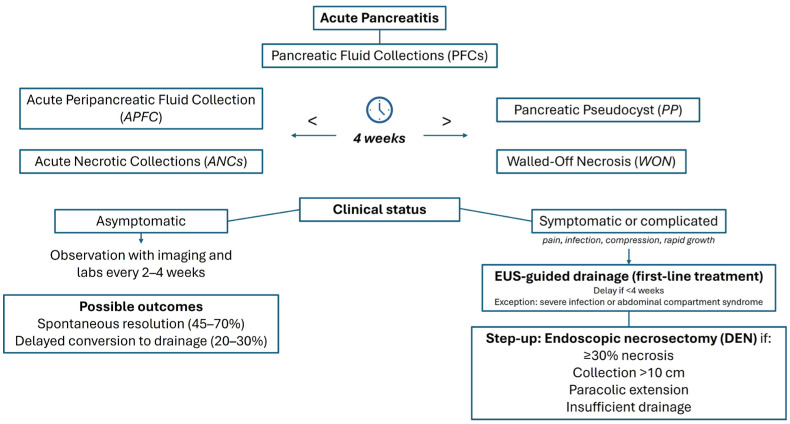
Proposed treatment algorithm for pancreatic fluid collections.

The optimal management of PFCs increasingly requires a personalized, patient-tailored approach (Figure 5).

Beyond anatomical considerations, factors such as the extent of necrosis, proportion of solid debris, underlying pancreatic ductal anatomy, and systemic condition (e.g., organ failure, comorbidities) are critical in guiding therapy. Advances in cross-sectional imaging, endoscopic techniques, and endoscopists’ training enable better characterization of collections, facilitating individualized strategies that balance efficacy with safety. In the future, personalized algorithms may integrate biomarkers, radiomic signatures, and machine-learning models to predict treatment response and guide modality selection.

## 9. Summary of Key Findings

The accumulated evidence consistently demonstrates that endoscopic drainage is superior to PD across most clinically relevant endpoints, including clinical success, need for reintervention, hospital stay, and mortality. Prospective and retrospective series (Wan et al., Keane et al., Samanta et al.) and the recent meta-analysis by Khizar et al. all confirm a clear advantage of the endoscopic route, particularly in WON with <40% solid components. While PD remains valuable in selected patients—particularly for collections not amenable to endoscopic access or as part of DMD—it is associated with longer hospitalization, higher risk of external fistula formation, and impaired quality of life. Based on current data, EUS-TD should be considered the first-line approach for symptomatic or infected PFCs, particularly WON. PD should be reserved for patients in whom endoscopic access is not technically feasible or as an adjunct in dual-modality therapy for extensive collections. Careful patient selection, multidisciplinary evaluation, and availability of advanced endoscopic expertise are key to optimizing outcomes. Despite clear progress, several questions remain unresolved. The optimal timing of intervention, the role of early versus step-up necrosectomy, and the long-term outcomes of novel stents remain areas of active investigation. Randomized studies comparing endoscopic, percutaneous, and hybrid strategies in clearly defined subgroups are urgently needed. The integration of biomarkers and predictive imaging into clinical decision-making will be critical to advancing truly personalized care.

## Figures and Tables

**Table 1:** Comprehensive MRI protocol for pancreatic pseudocyst and walled-off necrosis (WON). The table summarizes the main MRI sequences, their diagnostic purpose, and characteristic findings. MRCP = Magnetic Resonance Cholangiopancreatography; fat-sat = fat saturated; GRE = Gradient-Echo; DWI = Diffusion Weighted Imaging; ADC = Apparent Diffusion Coefficient.

Sequence	Purpose/Diagnostic Value
T2-weighted (axial and coronal, ±fat-sat)	Depicts fluid as high signal intensity (bright). Detects internal septations or debris as low-signal (dark) areas within fluid. Fat-suppressed T2 helps distinguish fluid from fat; necrotic fat remains hypointense on fat-sat T2.
MR Cholangiopancreatography (MRCP)	Heavily T2-weighted sequences optimized to visualize pancreatic and biliary ducts. Demonstrates ductal anatomy, communication between collections and the pancreatic duct, duct leaks, disconnected duct syndrome, or obstructive calculi.
T1-weighted (unenhanced)	Simple fluid appears hypointense (dark). Proteinaceous or hemorrhagic fluid shows intermediate to high signal. Useful for differentiating fluid composition.
Dynamic contrast-enhanced T1 (3D fat-sat GRE, arterial/portal/delayed phases)	Evaluates wall enhancement, internal septa, or solid components. Pseudocysts typically show thin rim enhancement, whereas WON walls are thicker. Non-enhancing internal material confirms necrotic debris.
Fat-saturated T1 (post-contrast)	Improves detection of subtle wall enhancement and hemorrhage. The fibrous capsule of mature collections usually enhances on delayed phases.
Diffusion-Weighted Imaging (DWI) (optional)	Identifies viscous/cellular debris and may suggest infection. Restricted diffusion (high DWI signal, low ADC) can correlate with infected or proteinaceous necrosis. Still emerging but complements CT signs (e.g., gas).

**Table 2:** Comparative Studies on Endoscopic Ultrasound guided drainage with Lumen Apposing Metal Stent (LAMS) vs. Double Pigtail Plastic Stent (DPPS).

Study	Design & Patients	Primary Endpoint	Main Results	Safety/AEs
Bang et al. [105]	RCT, n = 60 (LAMS = 31, DPPS = 29)	No. of procedures to achieve treatment success (clinical + CT at 6 months)	No significant difference (2 vs. 3, *p* = 0.192). Procedure time shorter with LAMS (15 vs. 40 min, *p* < 0.001). Higher costs with LAMS (USD 12,155 vs. 6609).	Stent-related AEs more frequent with LAMS (32.3% vs. 6.9%, *p* = 0.01): buried stents, severe bleeding, biliary strictures.
Boxhoorn et al. [106]	Multicenter prospective cohort vs. historical RCT cohort (n = 53 LAMS, n = 51 DPPS)	Requirement for ETN	ETN needed in 64% LAMS vs. 53% DPPS (NS). No differences in mortality, major AEs, LOS, or costs.	Bleeding: 9% LAMS vs. 22% DPPS (NS).
Karstensen et al. [107]	Single-center RCT, (n = 22 DPPS, n = 20 LAMS)	No. of necrosectomies to achieve treatment success (clinical + CT)	No significant differences: necrosectomies (2.2 vs. 3.2, *p* = 0.42), LOS (43 vs. 58 days, *p* = 0.71), mortality 4.8%.	AEs: 12% overall. DPPS group: perforations, sepsis. LAMS group: 1 sepsis.
Chen et al. [108]	Multicenter retrospective, n = 189 (LAMS = 102, DPPS = 87)	Clinical success (WON ≤3 cm within 6 months without PD or surgery)	Higher clinical success with LAMS (80.4% vs. 57.5%, *p* = 0.001). Surgery more frequent with DPPS (16.1% vs. 5.6%, *p* = 0.02). Recurrence lower with LAMS (5.6% vs. 22.9%, *p* = 0.04).	Overall AE rates comparable (9.8% LAMS vs. 10.3% DPPS). Severe AEs rare. Stent dysfunction similar (migration 2.9% vs. 6.9%, occlusion 20.6% vs. 12.6%).
Study	Design & Patients	Primary endpoint	Main results	Safety/AEs
Bang et al. [105]	RCT, n = 60 (LAMS = 31, DPPS = 29)	No. of procedures to achieve treatment success (clinical + CT at 6 months)	No significant difference (2 vs. 3, *p* = 0.192). Procedure time shorter with LAMS (15 vs. 40 min, *p* < 0.001). Higher costs with LAMS (USD 12,155 vs. 6609).	Stent-related AEs more frequent with LAMS (32.3% vs. 6.9%, *p* = 0.01): buried stents, severe bleeding, biliary strictures.
Boxhoorn et al. [106]	Multicenter prospective cohort vs. historical RCT cohort (n = 53 LAMS, n = 51 DPPS)	Requirement for ETN	ETN needed in 64% LAMS vs. 53% DPPS (NS). No differences in mortality, major AEs, LOS, or costs.	Bleeding: 9% LAMS vs. 22% DPPS (NS).
Karstensen et al. [107]	Single-center RCT, (n = 22 DPPS, n = 20 LAMS)	No. of necrosectomies to achieve treatment success (clinical + CT)	No significant differences: necrosectomies (2.2 vs. 3.2, *p* = 0.42), LOS (43 vs. 58 days, *p* = 0.71), mortality 4.8%.	AEs: 12% overall. DPPS group: perforations, sepsis. LAMS group: 1 sepsis.
Chen et al. [108]	Multicenter retrospective, n = 189 (LAMS = 102, DPPS = 87)	Clinical success (WON ≤3 cm within 6 months without PD or surgery)	Higher clinical success with LAMS (80.4% vs. 57.5%, *p* = 0.001). Surgery more frequent with DPPS (16.1% vs. 5.6%, *p* = 0.02). Recurrence lower with LAMS (5.6% vs. 22.9%, *p* = 0.04).	Overall AE rates comparable (9.8% LAMS vs. 10.3% DPPS). Severe AEs rare. Stent dysfunction similar (migration 2.9% vs. 6.9%, occlusion 20.6% vs. 12.6%).

**Table 3:** Comparison of Efficacy and Safety Across Treatment Approaches. EUS-TD, endoscopic ultrasound-guided transmural drainage; PD, percutaneous drainage; ESU, endoscopic step-up; SSU, surgical step-up.

Study	Design & Population	Comparison	Key Outcomes	Main Findings	Adverse Events (AEs)
Wan et al. [140]	8-year prospective cohort, n = 147 (62 EUS-TD, 67 PD)	EUS-TD vs. PD	Clinical success, reintervention, mortality	PP: EUS-TD higher initial success (94.9% vs. 65%, *p* = 0.003); fewer reinterventions (2.6% vs. 35%, *p* = 0.004). WON: initial success higher with EUS-TD (65.2% vs. 17%, *p* < 0.001).	PD had higher AEs (85.1% vs. 26.1%). Residual necrosis only in PD group. Mortality lower with EUS-TD (3 vs. 6 deaths).
Keane et al. [141]	14-year cohort, n = 164 (109 EUS-TD, 55 PD)	EUS-TD vs. PD	Treatment success, LOS, reintervention, residual collections	Treatment success higher with EUS-TD (70% vs. 31%, *p* < 0.001). PD needed more interventions (3.3 vs. 1.8), more residual collections (67% vs. 21%), more surgical rescue (11% vs. 4%). LOS shorter with EUS-TD (median 4 days).	Procedural AEs slightly higher in EUS-TD (10% vs. 1%) but outweighed by long-term benefits.
Samanta et al. [142]	Real-world cohort, India, n = 218 WON	EUS-TD vs. PD	Clinical success, impact of solid component	Clinical success higher with EUS-TD (92.1% vs. 64.6%, *p* < 0.0001). PD outcomes worse when >40% solid debris; EUS-TD best when <40%.	AE rates not detailed; safety of EUS-TD emphasized.
Khizar et al. [143]	Meta-analysis, 17 studies, n = 1170 (543 EUS-TD, 627 PD)	Pooled EUS-TD vs. PD	Clinical success, technical success, reintervention, LOS, mortality	Technical success similar. Clinical success favored EUS-TD (OR 2.23), especially WON (OR 2.74). LOS shorter (–15 days). Reinterventions fewer (OR 0.25). Mortality lower (OR 0.24).	AE rates lower with EUS-TD across pooled studies.
Bakker et al. [144]	RCT, n = 22	Endoscopic transgastric necrosectomy vs. surgical necrosectomy	IL-6, major complications/death	Composite endpoint lower with endoscopy (20% vs. 80%). Lower systemic inflammation.	Endoscopy prevented new-onset organ failure (0% vs. 50%) and reduced fistulas (10% vs. 70%).
Bang et al. [145]	RCT, single center, n = 66	ESU vs. minimally invasive SSU	Major complications/death, QoL, costs	ESU superior (11.8% vs. 40.6%, *p* = 0.007). Better QoL, lower costs.	Fewer complications with endoscopy; no fistulas vs. 28.1% in surgery.
TENSION trial [146]	RCT, multicenter, n = 98	ESU vs. SSU	Death/major complications (6 mo)	No difference in primary endpoint (43% vs. 45%). Endoscopy: shorter LOS, fewer ICU days, fewer fistulas.	Pancreatic fistulas fewer with endoscopy; mortality similar.
ExTENSION study [137]	Long-term follow-up of TENSION survivors, n = 83, 7-year follow-up	ESU vs. SSU	Death/major complications, reinterventions, pancreatic function	Primary endpoint similar (53% vs. 57%). Endoscopy: fewer fistulas (8% vs. 34%), fewer late reinterventions (7% vs. 24%), lower recurrent pancreatitis (19% vs. 37%).	Pancreatic insufficiency similar (≈40–60%). Fewer long-term complications with endoscopy.
Tan et al. [147]	Network meta-analysis, 21 studies, n = 1850	Endoscopic necrosectomy vs. step-up vs. surgical	Mortality, complications, organ failure, cost-effectiveness	Endoscopic necrosectomy ranked best across most outcomes; upfront open necrosectomy worst. Recovery faster (–34.1 days).	Lowest complication and fistula rates with endoscopy; economic benefit favored endoscopy.

## Data Availability

No new data were created or analyzed in this study. Data sharing does not apply to this article.
